# What Is the Optimal First Line Antiretroviral Therapy in Resource-Limited Settings?

**DOI:** 10.1371/journal.pmed.1001291

**Published:** 2012-08-14

**Authors:** Chris Kenyon, Robert Colebunders

**Affiliations:** 1 Division of Infectious Diseases and HIV Medicine, Department of Medicine, University of Cape Town, Observatory, Cape Town, South Africa; 2 University of Antwerp, Antwerp, Belgium; 3 Institute of Tropical Medicine, Antwerp, Belgium

## Abstract

A perspective by Chris Kenyon and Robert Colebunders discusses policy implications for use of first line antiretroviral therapies in resource-limited settings, emerging from a new research study conducted by Campbell and colleagues.

**Linked Research Article**

This Perspective discusses the following new study published in *PLoS Medicine*:

Campbell TB, Smeaton LM, Kumarasamy N, Flanigan T, Klingman KL, et al. (2012) Efficacy and Safety of Three Antiretroviral Regimens for Initial Treatment of HIV-1: A Randomized Clinical Trial in Diverse Multinational Settings. PLoS Med 9(8): e1001290. doi:10.1371/journal.pmed.10001290

Thomas Campbell and colleagues report findings of a randomized trial conducted in multiple countries regarding the efficacy of antiretroviral regimens with simplified dosing.

Choosing an optimal first line antiretroviral therapy (ART) in resource-limited settings (RLS) involves a careful balancing act. On the one hand, we would want to ensure that patients receive the most efficacious therapy available. On the other hand, the most efficacious regimens may be too costly for countries afflicted by the dual burdens of very high HIV prevalence and of resource scarcity. This dilemma is compounded by the recent emergence of good quality evidence to start ART earlier (i.e., at higher CD4 counts), which can improve life expectancy [1], reduce the risk of tuberculosis acquisition [1], and reduce the transmission of HIV [2]. These considerations will mean that about 9 million extra people will need to be placed on ART by 2015 [3]—all in the context of a decline in global funding for AIDS during 2009 and 2010 [3].

The World Health Organization (WHO) has thus advised that countries should consider a number of factors in addition to clinical efficacy when considering choice of ART. These include in-country ART costs, numbers of current and future individuals needing to start ART, and the country's national prevalence of chronic hepatitis B, tuberculosis, and anemia [4].

## PEARLS Contributions

In a new study published in this week's *PLoS Medicine*, Thomas Campbell and colleagues compared the efficacy and safety of three ART regimens for treatment naïve patients in the Prospective Evaluation of Antiretroviral Therapy in Resource-Limited Settings (PEARLS) trial [5]. They conducted a randomized controlled trial to compare the efficacy and safety of open-label ART with efavirenz plus lamivudine-zidovudine (EFV+3TC-ZDV), atazanavir plus didanosine-EC plus emtricitabine (ATV+DDI+FTC), or efavirenz plus emtricitabine-tenofovir-DF (EFV+FTC-TDF). Of note, their study population was considerably more representative of the global population of people with HIV than many other studies. The study population of 1,571 individuals with HIV-1 was drawn from nine countries (including eight low- and middle-income countries) in four continents. Forty-seven percent were women.

The trial found that ATV+DDI+FTC was inferior to EFV+3TC-ZDV in terms of treatment efficacy (21% and 15% treatment failures, respectively). EFV+3TC-ZDV and EFV+FTC-TDF were equally efficacious (19% and 18% treatment failures, respectively). This is in contrast to the only other randomized controlled trial to have evaluated the relative efficacy of these two regimens (GS-01-934), which found EFV+FTC-TDF to be more efficacious than EFV+3TC-ZDV [6]. In common with other trials, PEARLS found that the EFV+ZDV-3TC regimen was associated with more side effects requiring drug substitution than the EFV+FTC-TDF regimen.

How do we explain the discrepant findings between PEARLS and GS-01-934? The authors of PEARLS provide a plausible explanation relating to differences in how the primary efficacy endpoints were defined. GS-01-934 used as primary endpoint the US Food and Drug Administration (FDA) time to loss of virologic response (TLOVR). This includes all antiretroviral (ARV) substitutions as endpoints (failures). The ZDV containing arm had more adverse events than the TDF arm (22 versus 9), and this was the main determinant of the difference in primary endpoint between the two arms. Their explanation is given further credence by a reanalysis of PEARLS using the FDA-recommended TLOVR as the primary outcome. This transformed the PEARLS outcomes to concur with those of GS-01-934—the TDF regimen was superior.

In our opinion, a cogent case can be made that including protocol-pre-specified drug substitutions in an efficacy endpoint that is labeled as virological suppression is misleading. Both the rates of virological suppression and drug side effects are important endpoints, but it is unhelpful and misleading to conflate the two into one category and then label this as virological suppression. Of further note, the analytical approach taken in GS-01-934 favoured the commercial funder of the trial, Gilead Sciences. Six of the 12 authors of GS-01-934 were employees of Gilead, who manufacture TDF, and according to the authors of GS-01-934, “the study was designed by and the data were analyzed at Gilead Sciences” [6].

## Practical Implications

The PEARLS study provides further evidence to back up the first two of four ART regimens listed by the WHO guidelines as acceptable first line ART regimens: AZT +3TC+EFV, TDF+3TC (or FTC)+EFV, AZT+3TC+NVP, and TDF+3TC (or FTC)+NVP [4].

PEARLS confirms the widely held view that TDF/FTC/EFV is an excellent first line ART regimen for use in RLS [7]. Its advantages include daily dosing, availability in a fixed dose combination, compatibility with tuberculosis treatment, effect on hepatitis B, low rates of side effects, and more recently the fact that the cheapest generic price for TDF is lower than that of the equivalent for ZDV [3]. The ability of RLS to access the cheapest ARVs varies dramatically [3],[8]. The evidence from PEARLS that ZDV has equivalent virological suppression to TDF could be used by these countries and others to further link ZDV and TDF in competitive price reductions.

One of the most useful contributions that PEARLS makes to our knowledge of ART is the lack of heterogeneity of efficacy or toxicity of ART according to country, continent, race, and/or ethnicity. There have been numerous concerns raised in the literature that there may be increased risks of drug toxicity from particular ARVs in specific populations. It has been argued that there is a risk that both TDF [9] and EFV [10] would have greater side effect rates in African populations. Reassuringly, PEARLS found no evidence of differential efficacy or toxicity by country, continent, race, or ethnicity.

## Future Studies

Nevertheless, there are a number of limitations with the PEARLS trial that point the way for future research. One of these is the choice of protease inhibitor (PI)-based ART—unboosted ATV/DDI/FTC. A number of trials have shown the superiority of boosted- over unboosted-ATV, and this is now widely regarded as best practice [11]. A further problem is the inclusion of DDI, a drug whose toxicity and complexity in dosing has led to its exclusion from numerous guidelines for first line ART. It was thus not surprising that this regimen was less efficacious than EFV+3TC/ZDV. Given the suboptimal nature of this regimen, one certainly cannot generalize the findings to other PI-based ART. PI-based ART may be a good option for first line ART in RLS in persons who have been exposed to ARVs in the form of the prevention of mother-to-child transmission or pre-exposure prophylaxis [12]. Other first line regimens in need of further research in RLS are regimens including nevirapine 400 mg daily and rilpivirine (which has less neuropsychiatric side effects than EFV, and could possibly be produced for as little as US$10 per patient per year but is somewhat less effective in patients with high baseline viral loads [3]). New ARVs that may become options for the future include an alternative pro-drug version of tenofovir, GS-7340, which requires a lower dose and could be considerably cheaper than TDF [13]. PEARLS has provided policy makers and clinicians in RLS with reassurance of the equivalence, as far as virological suppression is concerned, of EFV+3TC-ZDV and EFV+FTC-TDF. For the reasons mentioned above, TDF containing ART will remain the optimal first line ART in RLS. Unfortunately, we remain a long way from ensuring that all ART-requiring patients in RLS receive optimal ART rather than more toxic regimens, or no ART at all.

## Abbreviations

ART: antiretroviral therapy
ARV: antiretroviral
PEARLS: Prospective Evaluation of Antiretroviral Therapy in Resource-Limited Settings
PI: protease inhibitor
RLS: resource-limited settings
TLOVR: time to loss of virologic response
WHO: World Health Organization
